# Feasibility and outcomes of atrial fibrillation screening using intermittent electrocardiography in a primary healthcare setting: A cross-sectional study

**DOI:** 10.1371/journal.pone.0198069

**Published:** 2018-05-24

**Authors:** Faris Ghazal, Holger Theobald, Mårten Rosenqvist, Faris Al-Khalili

**Affiliations:** 1 Karolinska Institute, Department of Clinical Sciences, Cardiology Unit, Danderyd Hospital, Stockholm, Sweden; 2 Karolinska Institute, Department of Neurobiology, Care Sciences and Society, Stockholm, Sweden; Universita degli Studi di Napoli Federico II, ITALY

## Abstract

**Background:**

Atrial fibrillation (AF) is a major risk factor for ischemic stroke unless treated with an anticoagulant. Detecting AF can be difficult because AF is often paroxysmal and asymptomatic. The aims of this study were to develop a screening model to detect AF in a primary healthcare setting and to initiate oral anticoagulant therapy in high-risk patients to prevent stroke.

**Methods:**

This was a cross-sectional study. All 70- to 74-year-old individuals registered at a single primary healthcare center in Stockholm were invited to participate in AF screening upon visiting the center during a ten-month period. Those who did not have contact with the center during this period were invited to participate by letter. Thirty-second intermittent ECG recordings were made twice a day using a handheld Zenicor device over a 2-week period in participants without AF. Oral anticoagulant therapy was offered to patients with newly detected AF.

**Findings:**

Of the 415 eligible individuals, a total of 324 (78.1%) patients participated in the study. The mean age of the participants was 72 years, 52.2% were female, and the median CHA2DS2-VASc score of the participants was 3. In the target population, 34 (8.2%) individuals had previously diagnosed AF. Among participants without previously known AF, 16 (5.5%) cases of AF were detected. The final AF prevalence in the target population was 12%. Oral anticoagulant therapy was successfully initiated in 88% of these patients with newly detected AF.

**Conclusions:**

The AF screening project exhibited a high participation rate and resulted in a high rate of newly discovered AF; of these newly diagnosed patients, 88% could be treated with an oral anticoagulant.

## Introduction

Atrial fibrillation (AF) is a common cardiac arrhythmia [1] and a frequent source of cardiac emboli leading to ischemic stroke [2] unless treated with an anticoagulant [3].

AF may be asymptomatic [4,5] and therefore often goes undiagnosed. Asymptomatic AF carries a high risk for ischemic stroke, similar to symptomatic AF [6]. Paroxysmal AF can be difficult to detect and is more prevalent than persistent AF in acute stroke [7]. Intermittent electrocardiogram (ECG) recordings can detect a high proportion of paroxysmal AF in patients with cryptogenic stroke, comparable to the proportion detected by five-day Holter monitoring [8]. Short-term ECG monitoring using a Zenicor thumb-ECG has a high sensitivity and specificity for detecting asymptomatic AF outside the hospital [9].

Although the prevalence of both AF and stroke increases with age [10], patients with paroxysmal AF are often younger than those with persistent AF [7]. Therefore, more extensive diagnostic effort is required to detect paroxysmal AF in younger patients.

Several studies [3,10,11] have reported the mean age of detected AF cases to be in the range of 70–73 years. Mass screening [12] for silent AF among 75- to 76-year-old individuals using intermittent ECG was shown to be feasible, although participation was relatively low. Therefore, effort is needed to increase the yield of AF screening, likely in primary healthcare and to screen people of relatively younger ages.

The purpose of the present study was to develop a screening model for primary healthcare settings using intermittent ECG to identify patients with AF and to initiate proper anticoagulant treatment in such patients to prevent future stroke.

The aims of this study were to assess the following:

1-Participation in AF screening in a primary healthcare center (PHCC) among individuals aged 70–74 years.
2-The total prevalence of both previously and newly diagnosed AF in the studied population.
3-The acceptability of initiating anticoagulant treatment for AF in this age group.
4-Clinical characteristics and the prevalence of cardiovascular risk factors for stroke and the management of modifiable risk factors.

## Methods

https://www.protocols.io/view/the-feasibility-and-outcome-of-atrial-fibrillation-m2fc8bn

### Design

Cross-sectional screening study.

### Study population

All individuals born between 1941 and 1945 who were registered at the Högdalen Primary Health Care Center in Stockholm during 2015.

### Inclusion criteria

All individuals in the target population who agreed to participate in the study.

### Screening period

From February 2015 to February 2016.

### Screening procedure

Individuals with previously known AF were invited for routine physician visits at the PHCC for follow-up according to national recommendations. Anticoagulant therapy was recommended to individuals with previously known AF who did not receive such treatment.

Individuals without previously known AF who visited the PHCC for health consultations during the inclusion period were invited to participate in the screening program. The remaining individuals who did not visit the PHCC during the first 10 months of the inclusion period received two written invitations to participate.

Participants received written and oral information about the study. All participants provided informed consent to participate by signing and submitting consent forms before entering the study. The responsible physician in the PHCC reviewed the medical histories of the participants, including their current medications, and performed a general medical examination that included blood pressure and fasting plasma glucose measurements. Participants without previously known AF were examined with a 12-lead electrocardiogram (ECG). If the ECG did not show AF, intermittent ECG recordings were performed for 30 seconds twice a day, and if palpitations were present, the ECG readings were performed for at least two weeks. An extended recording period was implemented in cases of infrequent recordings and suspected arrhythmia.

A Zenicor handheld ECG with an integrated mobile transmitter was used. When handheld ECG findings indicated AF or any other suspected pathological finding, the ECG was re-examined by an experienced cardiologist to confirm the diagnosis. Individuals with unclear or uninterpretable ECGs were further investigated using the Holter monitoring. An oral anticoagulant (OAC) was offered to patients with AF.

### Definition of the variables

AF was defined according to ESC guidelines [1] as a 30-second recording with an absolutely irregular rhythm without distinct p-waves.

The CHA2DS2-VASc score [1] was used to assess the risk of systemic thromboembolism. Medical records of the patients were used to evaluate cardiovascular morbidity among non-participants. Both the medical histories and medical records of the patients were used to determine cardiovascular and other non-cardiovascular morbidity among the participants.

Congestive heart failure (CHF) was defined based on the ESC guidelines as typical symptoms (e.g., breathlessness, ankle swelling and fatigue) that are caused by structural and/or functional cardiac abnormalities. Plasma NT-proBNP tests and echocardiography were used for the diagnosis of unclear cases.

For the diagnosis of hypertension, the following ESC guidelines were applied: brachial artery systolic blood pressure ≥140 mmHg and/or diastolic blood pressure ≥90 mmHg on at least two different occasions. Digital blood pressure measurements were made at rest in a sitting position.

Fasting plasma glucose ≥7.0 mmol/L on two different occasions was used as the criterion for a diagnosis of diabetes mellitus, according to recommendations of the World Health Organization.

Alcohol intake was assessed as the number of standard alcohol glasses consumed per week, where a standard alcohol glass contains 12 grams of pure alcohol.

The EQ visual analog scale [13], a standardized measure of health status developed by the EuroQol Group, was used as a quantitative measure of the participants’ self-health assessment. New York Heart Association functional classification was used to evaluate physical capacity.

### Statistical analyses

Categorical data were summarized by counts and percentages, whereas continuous data were described by the mean (with standard deviation) or the median (with interquartile range). Fisher’s exact test was used to analyze categorical variables. Student’s *t-*test or the Mann-Whitney test was used to compare continuous variables between two groups. Analysis of variance or the Kruskal-Wallis test was used to compare continuous variables among three groups. If these tests showed significant results, the Bonferroni or Dunn test was used as a post hoc test. Odds ratios with 95% confidence intervals were used to test for associations between AF and risk factors. For all tests, a probability value ≤0.05 was considered statistically significant. These analyses were performed using Stata statistical software version 10.

### Ethics approval

This study was performed in accordance with the Declaration of Helsinki and was approved by the Ethics Committee of Stockholm (DNR 2014/2061-31 and 2017/129-32).

## Results

### Participation

**Fig 1:**
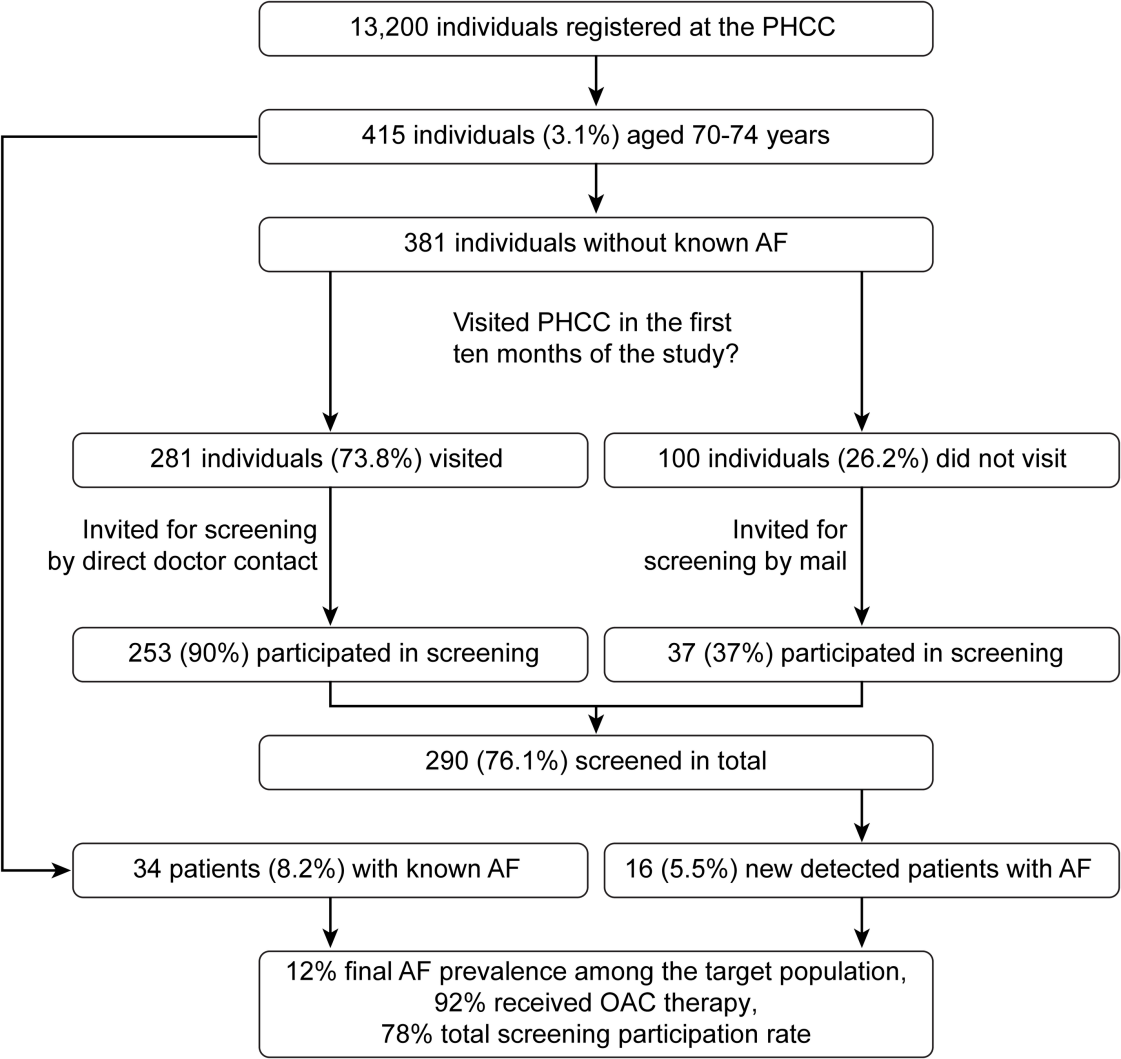
Flow chart illustrating the screening procedure.

Of the 13 200 individuals who were registered at the PHCC during 2015, 415 (3.1%) were aged 70–74 years (Fig 1). The corresponding proportion of the Swedish population in this age group was 5.2%. Women constituted 51.6% of the study population, while the corresponding proportion of women in the Swedish population in this age group was 51.2%.

**Fig 2:**
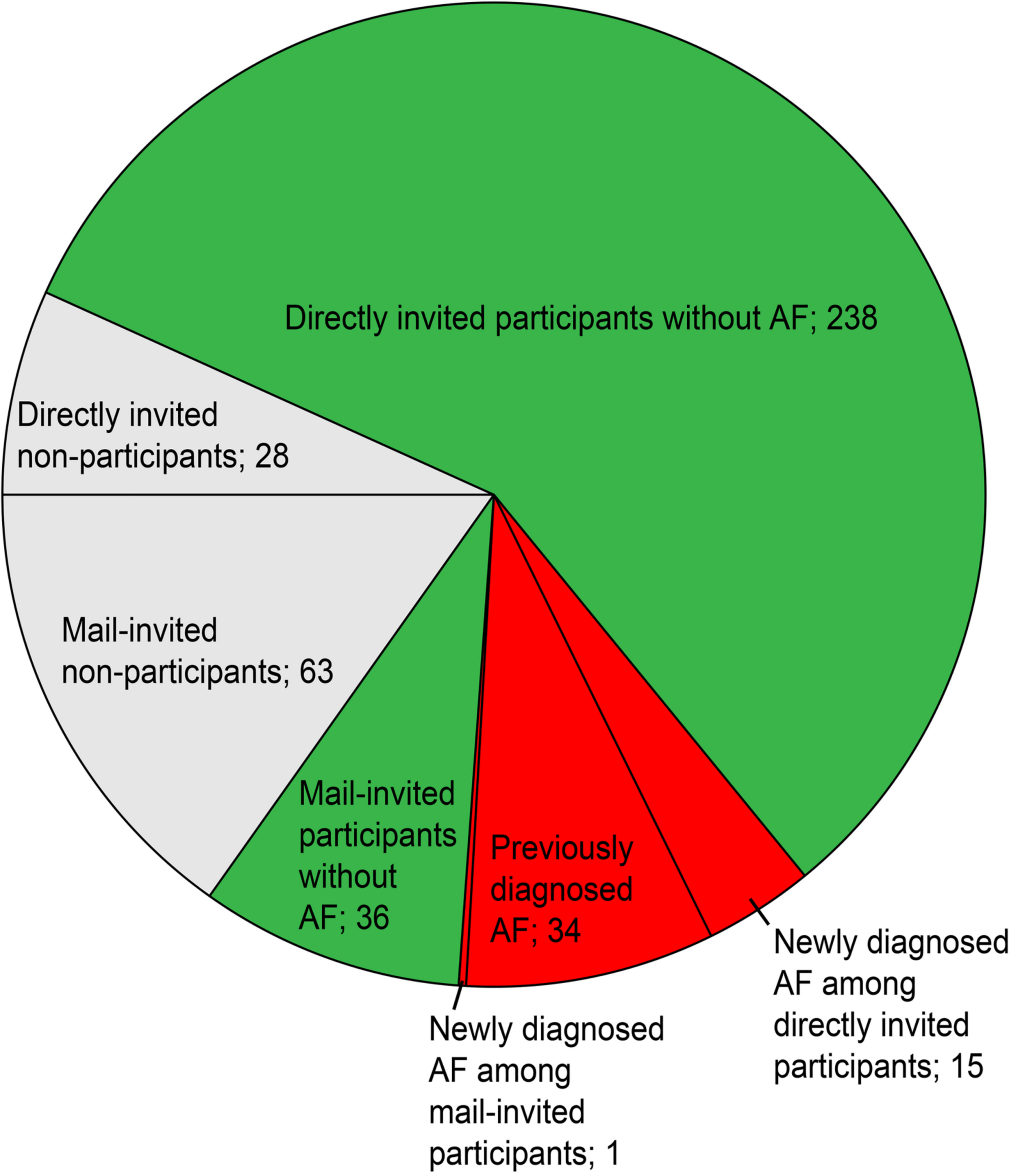
Screening results.

Of the 415 registered individuals, a total of 34 (8.2%) patients were already diagnosed with AF (Figs 1 and 2). Of the 381 individuals without previously known AF, 281 patients (73.8% of the target population) visited the PHCC for different health consultations during the first 10 months of the study. These patients were asked to participate in the AF screening. Among these patients, 253 (90%) participated in the screening, while 28 patients declined to participate. One hundred individuals did not visit the PHCC during the first 10 months of the study, and they were invited by mail to participate in the screening during the last two months of the study period. Of the mail-invited group, 37 individuals participated in the screening, six individuals declined participation, and 57 individuals did not respond.

The total participation rate was 78.1% (95% CI, 73.8–82). The prevalence of diabetes mellitus and hypertension was higher among participants than among non-participants (Table 1). The mean CHADS2-VASc score was 2.9 points for participants versus 2 points for non-participants. These differences were statistically significant. There was no statistically significant difference in age and sex between participants and non-participants.

**Table 1:** Demographic characteristics and morbidity of participants compared with non-participants.

	Participants, 324 (78.1% of target population)	Non-participants, 91 (21.9% of target population)	P-value
CHA2DS2-VASc score, mean (SD)	**2.9 (1.2)**	**2 (1)**	<0.001*
Diabetes mellitus, N (%)	**77 (23.8)**†	**7 (7.7)**	<0.001**
Hypertension, N (%)	**250 (77.2)**†	**27 (29.7)**	<0.001**
Congestive heart failure, N (%)	**22 (6.8)**	**0 (0)**	0.004**
Vascular diseases‡, N (%)	**41 (12.7)**	**5 (5.5)**	0.035**
Previous stroke, TIA or PTE, N (%)	30 (9.3)	4 (4.4)	0.096**
Women, N (%)	169 (52.2)	42 (46.2)	0.186**
Age (years), mean (SD)	71.9 (0.1)	72 (0.1)	0.564*

* Student’s *t-*test

† Evaluated by medical records, blood pressure measurements and fasting blood glucose levels

** Fisher’s exact test

‡ Myocardial infarction and/or peripheral artery disease

SD, standard deviation; TIA, transient ischemic attack; and PTE, peripheral thromboembolism.

### Screening results and OAC treatment

A total of 290 individuals, representing 76.1% of patients without previously known AF, were screened for AF. An intermittent ECG screening period of 14 days was implemented for 87% of the participants, and a 15- to 35-day screening period was implemented for the remaining participants. The median number of ECG recordings per individual was 39, with an interquartile range of 32–46.

Of the 290 individuals screened for AF, 16 (5.5%) new cases of AF were detected (95% CI, 3.2–8.8). Of these newly detected AF cases, 12-lead ECG performed at the index visit detected AF in three cases, corresponding to 1% of the screened individuals. The remaining newly detected AF cases were detected by handheld intermittent ECG. Ten of the newly detected AF patients had paroxysmal AF, while sex of those patients had non-paroxysmal AF. Of all newly detected AF patients, twelve (75%) had asymptomatic AF, and four exhibited AF-associated palpitations.

OAC therapy was initiated in 14 out of 16 (88%) newly detected AF patients. OAC therapy was contraindicated in one patient, and another patient refused the treatment. Among patients with previously known AF, six patients were not taking an OAC, and an OAC was subsequently given to four of these patients. OAC therapy was contraindicated in one patient, and another patient refused the treatment. Thus, anticoagulant therapy was initiated in a total of 18 patients. Therapy with a non-vitamin-K antagonist oral anticoagulant was initiated in 15 (83%) patients, and three (17%) patients were started on warfarin. Six participants without AF were already being treated with an anticoagulant for lung embolism, deep vein thrombosis or aortic thrombosis.

The final AF prevalence in the target population was 12% (95% CI, 9.1–15.6). Of all patients with AF, 92% received OAC therapy (95% CI, 80.8–97.8). One case of clinically significant supraventricular tachycardia and one case of clinically significant sinus bradycardia were identified. These patients were referred to a cardiac clinic for further evaluation.

Ten participants were further examined by Holter ECG for 24–120 hours. Six of these participants had frequent ventricular and/or frequent supraventricular ectopic beats without AF according to both handheld ECG and Holter ECG. Four participants had AF with short paroxysms that were first detected with handheld ECG and then confirmed with Holter ECG.

Among all participants, eight (2.5%) new cases of diabetes mellitus and 33 (10.2%) new cases of hypertension were detected. Among the 217 patients with known hypertension, twelve cases (5.5%) of severe hypertension (blood pressure ≥180/110 mmHg) were detected. Of these twelve patients, seven had not been receiving antihypertensive treatment. Hypertension was managed according to the national recommendations.

### Clinical characteristics of AF groups (S1 Table)

The diagnosis of sleep apnea was more prevalent among patients with newly diagnosed AF (18.8%) than among those without AF (1.8%; Table 2). The mean diastolic blood pressure was 6.5 mmHg higher (95% CI, 0.5–12.6) in patients with newly detected AF than in those without AF.

**Table 2:** Characteristics of participants without AF, patients with newly diagnosed AF and patients with known AF.

	Without AF, 274 persons	New AF, 16 patients	Known AF, 34 patients	P-value
Age, mean years (SD)	**71.9 (1.5)**	72 (1)	**72.6 (1.4)**	0.019*
Women, N (%)	148 (54)	7 (43.8)	14 (41.2)	0.299**
Non-Swedish birth country, N (%)	58 (21.2)	6 (37.5)	9 (26.5)	0.227**
Civil state living alone, N (%)	130 (47.4)	5 (31.3)	18 (52.9)	0.664**
Alcohol consumption, median glasses/week (IQR)	2 (0,6)	2 (0,7)	1 (0,5)	0.965***
Smoking: Current smoker, N (%)	48 (17.5)	1 (6.3)	6 (17.6)	0.470**
Previous smoker, N (%)	128 (46.7)	7 (43.7)	12 (35.3)	
Never smoker, N (%)	98 (35.8)	8 (50.0)	16 (47.1)	
CHA2DS2-VASc score, median (IQR)	**3 (2,3)**	**3 (2,3)**	**4 (3,5)**	0.001***^†^
CHA2DS2-VASc score, mean (SD)	**2.8 (1)**	**2.9 (1)**	**3.8 (1.8)**	<0.001*^†^
Congestive heart failure, N (%)	**7 (2.6)**	**1 (6.3)**	**14 (41.2)**	<0.001**^†^
Hypertension, N (%) post-screening	207 (75.5)	13 (81.3)	30 (88.2)	0.231**
Diabetes mellitus, N (%) post-screening	59 (21.5)	6 (37.5)	12 (35.3)	0.078**
Previous stroke and/or TIA, N (%)	**21 (7.7)**	**0 (0)**	**9 (26.5)**	0.003**^†^
Vascular disease^§^, N (%)	30 (10.9)	3 (18.85)	8 (23.5)	0.075**
Chronic obstructive pulmonary disease, N (%)	**26 (9.5)**	2 (12.5)	**9 (26.5)**	0.017**
Sleep apnea, N (%)	**5 (1.8)**	**3 (18.8)**	2 (5.9)	0.004**
Dementia, N (%)	**12 (4.4)**	**0 (0)**	**3 (8.8)**	0.200**
History of malignancy, N (%)	54 (19.7)	2 (12.5)	7 (20.6)	0.910**
Mobility class: No problems with walking	**218 (79.6)**	12 (75)	**18 (52.9)**	0.012**
Some problems with walking	**54 (19.7)**	4 (25)	**16 (47.1)**	
Bedridden	**2 (0.7)**	0 (0)	**0 (0)**	
Self-care class: No problems with self-care	**269 (98.2)**	15 (93.7)	**30 (88.2)**	0.011**
Some problems with self-care	**3 (1.1)**	1 (6.3)	**4 (11.8)**	
Inability to wash or dress self	**2 (0.7)**	0 (0)	**0 (0)**	
Class of usual activities (housework or leisure): No problems	**248 (90.5)**	14 (87.5)	**26 (76.5)**	0.004**
Some problems	**24 (8.8)**	1 (6.2)	**4 (11.7)**	
Inability to perform usual activities	**2 (0.7)**	1 (6.2)	**4 (11.7)**	
NYHA functional class: 1	**185 (67.5)**	8 (50)	**14 (41.2)**	0.001**
2	**83 (30.3)**	7 (43.7)	**14 (41.2)**	
3	**6 (2.2)**	1 (6.3)	**6 (17.6)**	
4	0 (0)	0 (0)	0 (0)	
Health assessment score, median (IQR)	**85 (75,90)**	88 (68,90)	**80 (60,85)**	0.003***
Body weight, mean kg (SD) women	**68.5 (13.5)**	72.4 (19.1)	**79.1 (9.5)**	0.017*
Body weight, mean kg (SD) men	88.1 (15.7)	95 (13.2)	91.9 (14.8)	0.295*
Height, mean cm (SD) women	161.2 (6.9)	161.7 (9.4)	161.2 (10.8)	0.983*
Height, mean cm (SD) men	175.7 (7.2)	176.4 (7.3)	177.7 (7.7)	0.496*
BMI, median kg/m2 (IQR) women	**26.0** **(22.9, 29.1)**	23.5 (22.6, 31.7)	**30.2** **(27.9, 32.9)**	0.010***
BMI, median kg/m2 (IQR) men	28.1 (25.9, 30.4)	31.5 (26.5, 34.4)	28.3 (26.6, 31.6)	0.311***
BP, mean mmHg (SD) systolic	**146.3 (21.8)**	**146.4 (15.8)**	**134.7 (17.4)**	0.011*^†^
BP, mean mmHg (SD) diastolic	**82.4 (11.9)**	**88.9 (13.1)**	85.9 (10.1)	0.033*
Beta blocker, N (%)	**82 (29.9)**	**6 (37.5)**	**30 (88.2)**	<0.001**^†^
Loop diuretic, N (%)	**16 (5.8)**	**5 (31.3)**	**12 (35.3)**	<0.001**^‡^
Angiotensin receptor antagonist, N (%)	64 (23.4)	5 (31.3)	10 (29.4)	0.552**
Angiotensin-converting enzyme inhibitor, N (%)	66 (24.1)	4 (25)	11 (32.4)	0.602**
Non-loop diuretic, N (%)	59 (21.5)	2 (12.5)	3 (8.8)	0.183**
Calcium antagonist, N (%)	56 (20.4)	5 (31.3)	11 (32.4)	0.169**
Statin, N (%)	87 (31.8)	7 (43.8)	14 (41.2)	0.349**
Antidiabetic drug, N (%)	46 (16.8)	4 (25)	10 (29.4)	0.173**
Acetylsalicylic acid, N (%)	55 (20.1)	5 (31.3)	3 (8.8)	0.133**
Anti-depressive agent, N (%)	26 (9.5)	1 (6.3)	2 (5.9)	0.912**

All variables with a significance level of p < 0.05 are presented in bold.

* ANOVA (Bonferroni test for statistically significant differences among groups)

** Fisher’s exact test

*** Kruskal-Wallis test (Dunn test for statistically significant differences among groups)

† Statistically significant difference between the known AF group and the new AF group as well as between the known AF group and the group without AF.

‡ Statistically significant difference between the group without AF and the new AF group as well as between the group without AF and the known AF group.

§ Myocardial infarction and/or peripheral artery disease; SD, standard deviation; IQR, interquartile range; TIA, transient ischemic attack; and BP, blood pressure.

Patients with known AF had CHF (41.2%) and a history of previous stroke or transient ischemic attack (26.5%) more often than both patients with newly detected AF and those without AF. Subsequently, patients with known AF had higher CHADS2-VASc scores, with a median of 4 points versus 3 points in the other two groups. These differences were statistically significant.

Patients with known AF were older than those without AF. The prevalence of chronic obstructive pulmonary disease and disabilities was higher among patients with known AF than among those without AF. Patients with known AF tended to feel worse, with a median health assessment score of 80% versus 85% in patients without AF. Women with known AF were more obese than women without AF. All these differences were statistically significant. In addition, the prevalence of hypertension, diabetes mellitus, vascular diseases and dementia was higher among patients with known than those without AF. However, these differences were not statistically significant.

### Discussion

This systematic screening study for AF using intermittent ECG by a family physician at the PHCC resulted in a high participation rate and detected many cases of AF, including asymptomatic and paroxysmal AF. Furthermore, OAC therapy was initiated at a high rate among patients diagnosed with AF. Diabetes mellitus and hypertension as risk factors for stroke were also screened and managed within the study.

### Participation

The overall participation rate in our study was 78.1%. Participation rates in previous studies that used intermittent ECG to screen for AF among 75- to 76-year-old patients were 53.8% [12] and 64% [14].

The participation rate was 57% in another study [15] that used a single ECG to screen for AF among patients aged 70 years or older; that study included patients who had been attended to by a general practitioner at least once in the past three years and did not have dementia or a terminal illness.

Thus, the participation rate in our study was higher than the participation rates in previous studies. In our study, 90% of patients directly contacted during visits to the PHCC participated, whereas 37% of patients indirectly contacted by mail participated. Therefore, opportunistic screening for AF by invitation when patients visit a PHCC may be a promising strategy to increase screening rates. Regular visitors of PHCCs are generally known to have chronic diseases and have a great need for healthcare. Such factors may result in better patient-physician relationships and greater adherence to the physician’s recommendations. Moreover, high cardiovascular morbidities are predictors for AF, thus increasing the probability of detecting new AF cases through opportunistic screening. Furthermore, patients not willing to participate seemed to be at a lower risk for stroke (Table 1).

### Screening outcomes and OAC treatment

The detection rate of new AF cases was 5.5% in this study. The detection rate of AF in a previous mass screening study [12] using the same intermittent ECG technique among 75- to 76-year-olds was 3%. This difference may be a result of higher morbidity among participants visiting the PHCC in our study.

The detection rate of AF by a single 12-lead-ECG screening in our study was 1%, and the prescreening AF prevalence was 8.2%. A previous opportunistic screening study [15] in general practice using a single ECG in patients with a median age of 77 years reported similar AF detection rates of 1% and prescreening AF prevalence of 9.3%. A community screening study [4] using a single ECG reported a total AF prevalence of 10.8% among 70- to 74-year-old patients.

Among the newly detected cases of AF in our study, 75% were classified as asymptomatic AF. A previous screening study [4] reported that 65.3% of new AF cases detected by a single ECG were asymptomatic. Asymptomatic AF recurrences were present in 49.5% of patients with AF [5].

In our study, 62.5% of newly detected AF cases were classified as paroxysmal AF. A previous screening study [14] reported that 75% of newly diagnosed AF cases were paroxysmal AF cases. Therefore, early detection of paroxysmal AF by intermittent ECG screening is essential.

OAC therapy was initiated in a high percentage (92%) of patients with newly diagnosed AF although most of the patients with newly detected AF were asymptomatic. A similar prescription pattern has been reported in a previous mass screening study [12].

Detection and management of both diabetes mellitus and hypertension as risk factors for stroke, were specifically considered in our study. Consequently, new cases of diabetes and new cases of hypertension were detected in 2.5% and 10.2% of patients, respectively. Treatment of hypertension might also decrease the risk for stroke.

### Clinical characteristics of the AF groups

The results of this study revealed that the newly detected AF cases were associated with sleep apnea, with an odds ratio of 12.4 (95% CI, 3.6–42.5). A previous cohort study [16] revealed that obstructive sleep apnea was an independent predictor of incident AF with a multivariate hazard ratio of 1.55 (95% CI, 1.21–2.00).

Diastolic blood pressure was significantly higher in new AF patients than in patients without AF. Undertreated hypertension may increase the risk of AF. A previous screening study [17] showed a significant association between detected AF cases and diastolic blood pressure, whereas the association of AF with systolic blood pressure was not significant.

Therefore, screening for AF in patients with sleep apnea or high diastolic blood pressure may be considered.

In our study, the median CHA2DS2-VASc score was 3 among new AF patients and those without AF, whereas the median CHA2DS2-VASc score was 4 among patients with known AF. Similar findings were reported in a previous mass screening study of 75- to 76-year-old patients. AF is usually paroxysmal in the early stages of the disease and then progresses to persistent and permanent AF with aging and associated comorbidities [18].

Thus, patients newly diagnosed with AF by screening appear to be as healthy as patients without AF. In contrast, patients with known AF are often older and more often have CHF and stroke histories. Within the narrow age range examined in our study, patients with known AF were older than those without AF. The odds ratios for CHF and stroke (and/or TIA) for patients with known AF were 26.7 (95% CI, 12.4–57.4) and 4.3 (95% CI, 1.9–9.9), respectively, compared with patients without AF. CHF itself may predict the progression of paroxysmal AF to permanent AF [1,18,19]. Therefore, early detection and management of AF may delay the progression to chronic AF with associated comorbidities.

In our study, chronic obstructive pulmonary disease was associated with known AF compared with no AF with an odds ratio of 3.4 (95% CI, 1.5–7.8). A previous study [20] reported that chronic obstructive pulmonary disease was independently associated with a two-fold higher risk of AF/atrial flutter.

Patients with known AF had more disabilities than the other groups. Health self-assessment score was lower (80%) among patients with known AF than among patients without AF (85%) in our study. This difference may be attributed to comorbidities and AF symptoms. A previous study [12] showed that the health self-assessment score was 67.1% among patients with controlled AF (sinus rhythm or AF ≤80 bpm) but 63.2% among patients with uncontrolled AF, and this difference was statistically significant.

AF was associated with body weight among women in our study. The association was statistically significant between body weight (and BMI) of women with known AF and women without AF. A previous screening study [12] showed that body weight was an independent risk factor for the development of AF. Body weight or obesity may even predict the progression of paroxysmal AF to permanent AF.

In our study, the prevalence of dementia was higher among patients with known AF than among those without AF. AF predicts dementia in patients with cognitive impairment [21]. Thus, evaluating cognitive function may be of value when detecting AF, given that patients with AF treated with oral anticoagulation are at lower risk for dementia than those without anticoagulation [22].

### Limitations

One limitation of this study is that the sample size was relatively small. Previous studies have reported associations of AF with sex, diabetes mellitus, hypertension and coronary artery disease. Our study also demonstrated associations of AF with these factors. However, the associations in our study were not statistically significant, likely because of the small sample size.

Our study was also a cross-sectional study. Therefore, it was difficult to establish a temporal relationship between AF and its risk factors (or its consequences), such as associations of AF with CHF or obesity.

Finally, this study was performed at a single PHCC. A multi-center screening study would be more representative and yield more generalizable results.

### Conclusion

Intermittent ECG screening for AF is feasible and effective and can have a high participation rate in PHCCs. Such screenings are also effective for initiating OAC therapy and managing cardiovascular risk factors to prevent stroke.

An interventional study is needed to further examine the effectiveness of such screening models in primary healthcare settings among patients 65 years and older and to establish whether these screening models can reduce the incidence of stroke.

## Supporting information

S1 Table Variables of the case reporting form. (XLSX)
